# Reduced Peritoneal NRF2 Immunoreactivity in Encapsulating Peritoneal Sclerosis and Its Implications for Redox Homeostasis

**DOI:** 10.3390/antiox15101234

**Published:** 2026-09-24

**Authors:** Tina Oberacker, Öztin Tüfek, Tobias Leibold, Adrian Salega, Rebekka Schier, Meng Dong, Leonie Kraft, Jörg Latus, Severin Schricker, Moritz Schanz

**Affiliations:** 1 Dr. Margarete Fischer-Bosch Institute for Clinical Pharmacology, Auerbachstr. 112, 70376 Stuttgart, Germanymeng.dong@ikp-stuttgart.de (M.D.); 2 University of Tübingen, Geschwister-Scholl-Platz, 72074 Tübingen, Germany; 3 Department of General and Visceral Surgery, Robert Bosch Hospital Stuttgart, Auerbachstr. 110, 70376 Stuttgart, Germany; t.leibold@medius-kliniken.de (T.L.); adrian.salega@rbk.de (A.S.); 4 Department of Internal Medicine and Nephrology, Robert Bosch Hospital Stuttgart, Auerbachstr. 110, 70376 Stuttgart, Germany; leonie.kraft@rbk.de (L.K.); joerg.latus@rbk.de (J.L.); severin.schricker@rbk.de (S.S.); moritz.schanz@rbk.de (M.S.)

**Keywords:** NRF2, oxidative stress, peritoneal dialysis, peritoneal membrane

## Abstract

Peritoneal dialysis (PD) is an effective modality of renal replacement therapy that provides patients with a high degree of autonomy. However, its long-term use is limited by the detrimental effects of PD fluids on the structure and function of the peritoneal membrane, ultimately leading to a loss of dialysis efficacy. Disruption of redox homeostasis plays a central role in these pathological changes, highlighting the importance of endogenous defense mechanisms in maintaining membrane integrity. Nuclear factor erythroid 2–related factor 2 (NRF2) is a key transcription factor regulating cellular antioxidant and cytoprotective responses. However, its immunoreactivity and localization in the peritoneal tissue of PD patients and patients with encapsulating peritoneal sclerosis (EPS) have not been well characterized. Therefore, we analyzed peritoneal biopsies from healthy controls, pre-dialysis patients, PD patients, and EPS patients. Immunohistochemical analysis demonstrated significantly reduced NRF2 immunoreactivity in patients with EPS compared with all other study groups. In contrast, nuclear factor kappa-light-chain-enhancer of activated B cells (NFκB) immunoreactivity was increased in PD and EPS patients. These results suggest that dysregulation of the NRF2–NFκB axis may be involved in peritoneal membrane injury, which warrants its further investigation as a potential target for preserving peritoneal membrane integrity during long-term PD.

## 1. Introduction

Peritoneal dialysis (PD) is an established modality of renal replacement therapy (RRT) that allows a substantial degree of patient autonomy. In the PD setting, the peritoneal membrane (PM) is continuously exposed to an unphysiological milieu through dialysis solutions, hyperosmolar glucose concentrations, and repeated episodes of oxidative stress [1].

These factors contribute to progressive pathophysiological alterations of the peritoneal membrane, including peritoneal fibrosis, angiogenesis, and mesothelial cell loss, which, in advanced stages, may ultimately culminate in encapsulating peritoneal sclerosis (EPS), leading to ultrafiltration dysfunction and ultimately technique failure [2,3]. Increasing evidence suggests that dysregulation of redox homeostasis plays a central role in these pathological changes [4].

Importantly, oxidative stress and chronic inflammation are not restricted to the peritoneal compartment but are characteristic features of chronic kidney disease (CKD) and progressively increase with declining kidney function. Uremic retention products, mitochondrial dysfunction, impaired antioxidant capacity, and activation of inflammatory pathways contribute to a persistent pro-oxidative and pro-inflammatory state in patients with advanced CKD and kidney failure [5]. These mechanisms may be further modulated by renal replacement therapy, with hemodialysis (HD) and PD being associated with distinct patterns of oxidative and inflammatory stress [6,7]. Thus, the systemic redox and inflammatory milieu present in uremic patients may also influence tissue-specific responses within the peritoneal membrane.

In this context, nuclear factor erythroid 2-related factor 2 (NRF2), a pivotal transcription factor involved in the regulation of cellular defense responses against oxidative stress via the induction of a broad spectrum of antioxidant and cytoprotective genes, has emerged as a focus of increasing scientific interest [8,9]. Under physiological conditions, NRF2 is constitutively degraded through Kelch-like ECH-associated protein 1 (KEAP1)-mediated ubiquitination [10]. In response to oxidative stress, KEAP1-mediated ubiquitination and degradation of NRF2 are inhibited, leading to NRF2 stabilization and accumulation, followed by its translocation into the nucleus, where it induces the transcription of numerous antioxidant and cytoprotective genes, including heme oxygenase-1 (HO-1), NAD(P)H quinone oxidoreductase 1 (NQO1), superoxide dismutase (SOD), catalase, and glutathione-related enzymes [11].

The NRF2 pathway has also been implicated in the pathophysiology of CKD. Experimental studies have demonstrated impaired NRF2-KEAP1 signaling and reduced antioxidant defense in chronic renal failure [12]. Clinical studies further suggest that NRF2-related responses may differ according to CKD stage and renal replacement therapy. In nondialysis CKD patients, NRF2 expression was comparable with that of healthy controls, whereas hemodialysis patients showed reduced NRF2 expression accompanied by increased NFκB expression [6]. Similarly, reduced NRF2 and NQO1 expressions, together with increased NFκB expression, have been reported in patients undergoing hemodialysis [7]. These findings suggest that NRF2-dependent antioxidant responses may vary with CKD stage and renal replacement modality.

In parallel, nuclear factor kappa B (NFκB), a central regulator of inflammatory gene expression, is activated in CKD and contributes to the chronic inflammatory phenotype associated with renal dysfunction and uremia [7,13]. Crosstalk between NRF2-dependent antioxidant pathways and NFκB-mediated inflammatory signaling has been described, suggesting that the balance between these pathways may be relevant to the progression of CKD-associated tissue injury [12,14]. Studies in patients receiving renal replacement therapy have further indicated that oxidative stress and inflammatory signaling remain altered during dialysis, with differences between HD and PD suggesting that the dialysis modality may influence systemic redox and inflammatory responses [15,16].

Various experimental studies suggest that NRF2 activation attenuates peritoneal fibrosis and may help preserve peritoneal membrane function during long-term PD [17,18,19,20,21,22]. In addition, inflammatory signaling pathways, including NFκB, have been implicated in mesothelial injury, inflammation, and the development of peritoneal fibrosis during chronic PD exposure [23,24]. These findings suggest that an imbalance between antioxidant defense mechanisms and pro-inflammatory signaling may contribute to progressive peritoneal membrane injury.

Despite these promising experimental findings, clinical data on the expression and subcellular localization of NRF2 in human peritoneal tissue remain limited, particularly in patients receiving PD and in those with EPS.

In particular, it remains uncertain how NRF2 expression and localization in the peritoneal membrane relate to the systemic uremic state and to pathological changes occurring during PD and how these compare with the inflammatory signaling environment associated with NFκB. Therefore, in this study, peritoneal biopsy samples from healthy controls, uremic patients prior to dialysis initiation, PD patients, and patients with EPS were examined to assess the expression and localization of NRF2, as well as its potential role in maintaining the integrity of the peritoneal membrane.

## 2. Materials and Methods

### 2.1. Chemicals

The chemicals used in this were obtained from Sigma-Aldrich (Taufkirchen, Germany) unless otherwise indicated.

### 2.2. Ethical Statement

This study was conducted in accordance with the Declaration of Helsinki and approved by the Ethics Committee of the Eberhard-Karls University Tübingen, Germany (process number: approval number 322/2009BO, approval date: 14 October 2009; approval number 317/2019BO1, approval date: 18 September 2019, updated 4 September 2024 and approval number 609/2019BO2, approval date: 16 September 2019, updated 8 July 2024). Written informed consent was obtained from all subjects involved in the study.

### 2.3. Study Cohort and Study Design

A part of the patient cohort included in this study (*n* = 126) has been described previously [25]. The earlier publication reported the patients’ baseline characteristics as well as data on submesothelial thickness, mesothelial cell loss, and the immunohistochemical expression of NFκB. The current study addresses a distinct research question and is based on a substantially expanded cohort of 191 patients/biopsies, including 65 newly added patients/biopsies. The newly added cases are distributed across different clinical subgroups, with all 14 patients in the EPS subgroup being newly included; the previous publication did not include patients with EPS.

Selected data reported in the previous study are presented again to provide a comprehensive characterization of the expanded cohort and to serve as a reference for the newly included patients and analyses. This approach enables direct comparison across the entire study population and facilitates the interpretation of any novel findings.

Although a part of the patient cohort overlaps with the cohort described published study, all representative images shown in the present manuscript were newly acquired and are independent of those published previously. Therefore, no previously published figures have been reused.

### 2.4. Inclusion and Exclusion Criteria

This study included adult patients with advanced chronic kidney disease (CKD stages 4 and 5) who underwent surgery, as well as healthy control subjects undergoing elective abdominal surgery. Patients younger than 18 years of age and those with diabetes mellitus or acute peritonitis were excluded, as both conditions are associated with alterations in oxidative stress and inflammatory status and could therefore confound the study *outcomes*.

### 2.5. Patient Subgroups Within the Study Cohort

Patients were assigned to one of the following four groups:

Healthy controls with normal kidney function (estimated glomerular filtration rate, eGFR) > 60 mL/min/1.73 m^2^); uremic patients with advanced chronic kidney disease (eGFR < 20 mL/min/1.73 m^2^) prior to the initiation of dialysis therapy; patients receiving peritoneal dialysis (PD); and patients undergoing long-term peritoneal dialysis with clinical evidence of encapsulating peritoneal sclerosis (EPS).

EPS was diagnosed according to established clinical, radiological, and histopathological criteria as described by Nakamoto et al. [26], Vlijm et al. [27], and Honda et al. [28], respectively.

### 2.6. Peritoneal Biopsies

Peritoneal biopsies were obtained from the Peritoneal Biopsy Registry of Robert Bosch Hospital, Stuttgart, Germany. Samples from the healthy control group were collected during various elective surgical procedures (e.g., cholecystectomies or herniotomies). In uremic patients, peritoneal biopsies were taken during the insertion of the CAPD catheter. In PD patients, biopsies were obtained during the removal of the CAPD catheter or during surgical correction of a misplaced catheter. In patients with EPS, biopsies were obtained as diagnostic biopsies in cases of clinical suspicion of EPS.

Overall, 191 patients with corresponding peritoneal biopsies were included in the study, comprising 34 healthy controls, 69 uremic patients, 74 PD patients, and 14 patients with EPS. Due to structural characteristics and differences in tissue availability, not all biopsies were suitable for every analysis. Consequently, the number of evaluable biopsies varied between the individual analyses.

Regarding biopsy localization, biopsies were obtained from the parietal peritoneum in 162 cases and from the visceral peritoneum in 2 cases. For 27 biopsies, the exact anatomical localization was not documented. These numbers totaled 191 biopsies.

Table 1 summarizes the clinical characteristics and descriptive statistics of the study cohort.

### 2.7. Pathology

All biopsy samples were fixed in 4% formalin and paraffin-embedded according to established standard procedures. For morphological assessment, 3 μm tissue sections were routinely prepared and stained with hematoxylin and eosin (HE) as previously described [25].

For analysis, all sections were visualized and digitally scanned using an Olympus VS120 automatic slide scanner in conjunction with a BX61VS microscope fitted with an UPLSAPO 40× objective (Olympus, Hachioji, Japan).

### 2.8. Mesothelial Cell Loss and Mean Submesothelial Thickness

Mean thickness of the submesothelial layer and mesothelial cell loss were evaluated using the imaging software OlyVIA (Version 3.3; Olympus). Submesothelial thickness was calculated by measuring the thinnest and thickest areas of the layer and determining the average of both values [29].

Mesothelial cell loss was categorized as intact (0), partially absent (1), or completely absent (2).

### 2.9. Immunohistochemistry (IHC)

Immunohistochemistry was performed as described in previous publications [25,30]. Dewaxed and rehydrated tissue sections were pretreated in a steamer, using an antigen retrieval solution (NRF2: without pre-treatment; NFkB staining: pH 6, S 1699; DAKO Glostrup, Denmark, 30 min). A negative control (without primary antibody) and a positive control were included in each staining (Supplementary Figure S1).

The following antibodies were used: NRF2 polyclonal rabbit antibody, (16396-1-AP; Proteintech, Rosemont, IL, USA) diluted 1:500 in antibody diluent (S 2022, DAKO, Glostrup, Denmark), and NFκB polyclonal rabbit antibody (sc-109; Santa Cruz Biotechnology, Dallas, TX, USA), diluted 1:200 in antibody diluent (S2022; DAKO, Glostrup, Denmark [25]). Kidney and lymph node were selected as positive controls for the detection of NRF2 and NFκB, respectively.

For analysis, all sections were visualized and scanned using an Olympus VS120 automated slide scanner fitted with a BX61VS microscope (objective: UPLSAPO 40×; Olympus).

### 2.10. Evaluation of Histo-Score

Immunohistochemical results were evaluated semi-quantitatively as previously reported [25,30]. To avoid bias, the Histo-Score assessment was performed in a blinded manner with respect to the respective clinical groups. The analysis included the following tissue components: mesothelium, submesothelium, fatty tissue, and vascular endothelium. Each component was assessed separately by calculating the product of staining intensity and the proportion of positively stained cells. The final Histo-Score was obtained by summing the four calculated values.

Color intensity was graded as follows: no staining (0), weak (1), moderate (2), or strong (3).

The percentages of positive cells were categorized manually by experienced members of the study team.

For NRF2- or NFκB-positive cells, the distribution was as follows: <1% (0), 1–10% (1), 10–25% (2), 25–50% (3), 50–75% (4), and >75% (5) [25]. For NRF2, nuclear and cytoplasmic staining were not separately assessed or scored; the Histo-Score therefore reflects overall NRF2 immunoreactivity.

### 2.11. Multiplex Immunofluorescence Staining and Image Analysis

Seven-color multiplex immunofluorescence staining was performed using the OPAL^TM^ multiplexing method (simultaneous detection of 6 antibodies). The sections were deparaffinized, rehydrated, and subjected to heat-induced epitope retrieval (antigen retrieval solution, pH 9, S 2367; DAKO, Glostrup, Denmark, 30 min). After cooling and washing with deionized water and the washing buffer (25 mM Tris-HCI, pH 7.5 150 mM NaCl 0.05% Tween20 (*v*/*v*)) for 5 min each, all sections were incubated for 10 min in a peroxidase blocking solution (S 2023; Agilent, Waldbronn, Germany) to block endogenous peroxidases. Subsequently, all slides were incubated with the first primary antibody (at room temperature for 30 min). Before incubation with the secondary antibody mix using the Multiplex Opal IHC-Kit (NEL861001KT, Akoya Biosciences, Berlin, Germany) at room temperature for 30 min, all sections were washed three times for 2 min with the washing buffer. Afterwards, all slides were incubated with the secondary antibody (at room temperature for 10 min) and washed three times for 2 min with the washing buffer. The antibodies were visualized with a fluorescent tyramide using the Opal Polaris 7-Color Manual IHC Kit (NEL861001KT, Akoya Biosciences; at room temperature for 10 min each). After washing three times with the washing buffer, the epitope retrieval (30 min, pH 6) and staining were repeated sequentially for different primary antibodies and fluorescent tyramide combinations (at room temperature for 10 min each, DIG-Opal 780 at room temperature for 60 min). Finally, the slides were incubated with DAPI for 5 min at room temperature, washed with washing buffer for 2 min, and mounted with ProLong^®^ Diamond Antifade Mountant (P36961, Thermo Fischer Scientific, Ettlingen, Germany).

The following primary antibodies were used: αSMA (M0851, DAKO) with 1:200 dilution, NRF2 (16396-1-AP; Proteintech, Rosemont, IL, USA) with 1:500 dilution, CK18 (M7010, DAKO, Glostrup, Denmark) with 1:50 dilution, and CD31 (clone 89C2, Cell Signaling Technology, Danvers, MA, USA) with 1:800 dilution.

The multiplex-stained slides were imaged using a PhenoImager Fusion system (Akoya Biosciences). After scanning the images, spectral unmixing of whole tissue sections was performed in inForm (Version 3.1.0 https://www.quanterix.com/software-data-analysis-solutions/inform-software/, Akoya Biosciences, Marlborough, MA, USA, accessed on 7 September 2026) using tissue-specific autofluorescence controls. The unmixed images were stitched using Visiopharm (version 2026.08.1 x64, https://visiopharm.com/product-releases/visiopharm-software-updates-2025-08-1/, accessed on 7 September 2026) and analyzed using QuPath29 (version 0.6.0, https://github.com/qupath/qupath/releases/tag/v0.6.0, accessed on 7 September 2026). Nuclei were detected with Cell Detection and the StarDist extension (Version v0.6.0, https://github.com/qupath/qupath-extension-stardist/releases/tag/v0.6.0, accessed on 7 September 2026). The percentage of cells stained positive for a marker was calculated. The images were visually reviewed to ensure reliable phenotyping.

### 2.12. Statistical Analysis and Data Handling

To compare the different subgroups, the non-parametric Kruskal–Wallis test followed by Dunn’s post hoc multiple-comparison test was used. As the statistical analyses were based on non-parametric tests, no assumption of normally distributed data was required. Data are presented as median with interquartile range (IQR) for clinical parameters and as mean ± SEM for experimental data, as indicated in the respective figure legends. Statistical analyses were performed using GraphPad Prism 10 (GraphPad Software Inc., San Diego, CA, USA). All statistical tests were two-sided, and a *p*-value < 0.05 was considered statistically significant; *p* ≥ 0.05 was considered non-significant. No correction for multiple testing beyond Dunn’s post hoc procedure was applied.

## 3. Results

To investigate the expression and localization of NRF2, as well as its potential role in maintaining the integrity of the peritoneal membrane, we collected peritoneal biopsies from patients in our extensive PD and EPS registries.

The cohort comprised four groups: controls with normal renal function, uremic patients who had not yet started dialysis, PD patients, and long-term PD patients exhibiting signs of EPS. Although the overall sex distribution was relatively balanced, an equal representation of males and females could not be achieved across all subgroups. The control group contained considerably more women than men, whereas the uremic subgroup comprised nearly twice as many men as women. The most pronounced imbalance was observed in the EPS subgroup, where male participants outnumbered female participants by approximately three to one.

However, age distribution was generally comparable between the four subgroups. The median ages were 52.0 years (IQR: 35.8–62.3) for controls, 59.0 years (IQR: 46.5–67.0) for uremic patients, 57.0 years (IQR: 43.8–65.5) for PD patients, and 50.0 years (IQR: 39.8–56.3) for participants in the EPS group. The median BMI values differed slightly between the study groups. The control group had a median BMI of 26.7 kg/m^2^ (IQR: 25.6–28.7), compared with 25.6 kg/m^2^ in pre-dialysis patients (*p* = 0.6845), 25.1 kg/m^2^ in PD patients (*p* = 0.1731), and 22.5 kg/m^2^ in the EPS group (*p* = 0.1352) (Table 1).

A substantial difference in hypertension prevalence was observed across the study groups. As expected, hypertension affected 15% of healthy controls, whereas the corresponding rates were 84% in the uremic group, 76% in the PD group, and 93% in the EPS subgroup. Regarding smoking status, 5% and 7% active smokers were identified in the control and pre-dialysis groups, respectively. The PD group included 12% active smokers, and 14% active smokers were present in the EPS subgroup (see Table 1).

The median dialysis duration was 32.0 months (IQR: 11.0–53.5) among PD patients and 70.0 months (IQR: 52.8–108.0) (*p* > 0.9999) among patients with EPS.

Regarding laboratory parameters, with the exception of creatinine and urea, no notable abnormalities were observed for leucocytes and hemoglobin in the study cohort. The clinical characteristics and descriptive statistics of all study participants are summarized in Table 1.

### 3.1. Structural Changes to the Peritoneal Membrane Associated with Peritoneal Dialysis

As demonstrated by our previous studies, the initiation of peritoneal dialysis is associated with structural changes to the peritoneal membrane [4,25,29].

To investigate pathological changes of the peritoneal membrane associated with peritoneal dialysis over time, submesothelial thickness and mesothelial cell loss were analyzed. Uremic patients showed no significant differences compared with controls (153.3 ± 8.8 μm vs. 142.7 ± 28.0 μm; not significant) (Figure 1A,B). By contrast, the biopsy specimens from PD and EPS patients demonstrated a pronounced increase in mean submesothelial thickness relative to both controls (315.6 ± 30.5 μm and 631.0 ± 111.1 μm vs. 142.7 ± 28.0 μm; *p* < 0.0001) and uremic patients (315.6 ± 30.5 μm and 631.0 ± 111.1 μm vs. 153.3 ± 8.8 μm; *p* < 0.0001) (Figure 1A,B). In addition, mesothelial cell loss was significantly elevated in PD and EPS patients compared with the control and pre-dialysis groups (*p* < 0.0001) (Figure 1A,C).

### 3.2. Dysregulation of the NRF2–NFκB Axis in Encapsulating Peritoneal Sclerosis

It is well known that PD patients show increased levels of oxidative stress in the peritoneal membrane [4,25]. For this reason, maintaining a stable redox homeostasis is extremely important. Among other factors, the transcription factors NRF2 and NFκB are involved in the regulation of cellular responses to oxidative stress and inflammation. Experimental studies have shown that NRF2 primarily mediates cell-protective and antioxidant defense mechanisms to limit tissue damage associated with oxidative stress. As the localization and immunoreactivity of NRF2 in the peritoneal membrane of long-term PD patients have not been adequately characterized, immunohistochemistry for NRF2 was performed.

The immunohistochemical analysis demonstrated that NRF2 immunoreactivity was predominantly localized to the mesothelium and vascular endothelium. Additional staining was observed in the submesothelium, whereas only minimal expression was detected in adipose tissue (Figure 2A and Supplementary Figure S2). Overall, NRF2 immunoreactivity was markedly stronger than that of the other markers examined.

Semi-quantitative assessment using the Histo-Score revealed a significant reduction in NRF2 immunoreactivity in patients with EPS compared with all other study groups (Figure 2B).

NFκB is a key regulator of pro-inflammatory signaling pathways and promotes the transcription of cytokines and mediators involved in chronic inflammation.

In the PD setting, alterations in the NRF2/NFκB balance may be associated with structural and inflammatory changes to the peritoneal membrane. To further characterize this potential association, we also stained our peritoneal biopsies for NFκB. Positive NFκB staining was identified in mesothelial cells, the submesothelium, and endothelial cells of blood vessels, whereas no immunopositive cells were observed within adipose tissue (Figure 3A and Supplementary Figure S2). Based on the Histo-Score evaluation, NFκB immunoreactivity remained low in the control and uremic tissue samples. However, a marked increase in NFκB-positive cells was observed in samples from patients undergoing dialysis. This increase was even more pronounced for EPS patients, showing a significant difference compared with the PD group (Figure 3B).

To further characterize the cellular localization of the observed changes in NRF2 immunoreactivity, multiplex immunofluorescence staining was performed using cell-specific markers in combination with NRF2 (Figure 4 and Supplementary Figures S3 and S4). Co-localization of NRF2 was observed with CK18, αSMA, and CD31, indicating that NRF2 is present in mesothelial, submesothelial, and vascular endothelial cell populations. Furthermore, analysis of the small biopsy specimens included in the multiplex analysis corroborated the IHC findings. NRF2 immunoreactivity was markedly higher in PD than in EPS (Figure 4 and Supplementary Figure S3).

## 4. Discussion

Peritoneal dialysis is associated with increased oxidative stress within the peritoneal membrane, underscoring the importance of maintaining redox homeostasis for membrane integrity [4,25]. Key regulators of this balance include the transcription factors NRF2 and NFκB, which govern cytoprotective antioxidant responses and inflammatory signaling, respectively. Given the established functional interplay between these pathways, alterations in their expression and activity may be associated with peritoneal injury. However, NRF2 immunoreactivity and localization in the peritoneal membrane of long-term PD patients have not been systematically characterized. Therefore, NRF2 immunoreactivity was evaluated via immunohistochemistry in the present study.

Our findings demonstrate significantly reduced peritoneal NRF2 immunoreactivity in patients with EPS compared with all other study groups. Reduced NRF2 immunoreactivity may reflect impaired alterations in local antioxidant responses in advanced peritoneal injury and may be associated with persistent oxidative stress and fibrotic changes. In contrast, NFκB immunoreactivity showed a different pattern. While NFκB immunoreactivity remained low in control and uremic tissue samples, it was increased in samples from patients undergoing PD and was further increased in samples from patients with EPS. The more pronounced increase in NFκB immunoreactivity compared with the reduction in NRF2 immunoreactivity may indicate that additional regulatory mechanisms are involved in the inflammatory response observed in advanced peritoneal injury.

Our findings are consistent with previous evidence demonstrating progressive disturbances in redox homeostasis across the course of CKD and during renal replacement therapy. Oxidative stress has been shown to increase with advancing stages of CKD, indicating that an altered redox balance is already present before the initiation of dialysis [5,31]. In patients receiving renal replacement therapy, hemodialysis and peritoneal dialysis have been associated with increased oxidative stress and alterations in antioxidant status compared with healthy controls [32]. In PD patients, oxidative stress has further been linked to chronic inflammation, peritoneal fibrosis, loss of ultrafiltration capacity, and technique failure [33]. These systemic and dialysis-associated alterations provide an important context for our findings in peritoneal tissue.

Importantly, most previous clinical studies have assessed systemic circulating markers of oxidative stress or inflammatory signaling rather than NRF2 and NFκB immunoreactivity directly within the human peritoneal membrane. In the present study, NRF2 immunoreactivity was significantly reduced in EPS patients, whereas NFκB immunoreactivity was increased in PD patients and further increased in EPS patients. Thus, our findings extend previous observations of systemic redox and inflammatory alterations by demonstrating distinct local changes in NRF2 and NFκB immunoreactivity within peritoneal tissue from patients with different degrees of peritoneal injury.

Previous clinical studies have demonstrated alterations in the NRF2–NFκB axis in patients with advanced CKD and during dialysis. Leal et al. reported that NRF2 and NFκB mRNA expression in nondialysis CKD patients was comparable with that of healthy individuals, whereas hemodialysis patients showed reduced NRF2 and increased NFκB expression in peripheral blood mononuclear cells [6]. Similarly, Pedruzzi et al. demonstrated reduced Nrf2 and increased NFκB expression in peripheral blood mononuclear cells from hemodialysis patients, accompanied by increased systemic markers of oxidative stress and inflammation [7].

These findings suggest that the NRF2–NFκB balance may be relatively preserved in nondialysis CKD patients, whereas greater dysregulation has been observed in patients receiving hemodialysis.

In PD, NFκB-related inflammatory signaling has also been associated with peritoneal membrane function. In a longitudinal study of incident PD patients, levels of NFκB-related downstream mediators, including IL-6 and COX-2, were associated with peritoneal transport characteristics and were increased in patients experiencing peritonitis [16]. In our study, NRF2 and NFκB immunoreactivity did not differ significantly between uremic and control peritoneal tissue samples, whereas more pronounced alterations were observed in samples from patients undergoing PD, and particularly patients with EPS. These findings are broadly consistent with previous clinical evidence of altered redox and inflammatory signaling during dialysis but extend these observations by demonstrating altered NRF2 and NFκB immunoreactivity directly within human peritoneal tissue.

These findings are consistent with previous studies investigating PD-associated inflammation and fibrosis. Topley and colleagues established the peritoneum as an active immunological compartment capable of generating a local cytokine network, with early activation of NFκB-dependent inflammatory pathways in response to peritoneal dialysis solutions [34,35]. Krediet and colleagues further demonstrated that long-term PD is associated with progressive structural and functional alterations of the peritoneal membrane, which are linked to chronic inflammation and repeated injury. In this context, peritoneal inflammation represents a dynamic and time-dependent process that may evolve from an early cytokine-rich phase to a late-stage fibrotic phenotype characterized by reduced cellular activity in advanced encapsulating peritoneal sclerosis [35,36].

Several NRF2-activating compounds have demonstrated anti-inflammatory and cytoprotective effects in preclinical models of oxidative stress and fibrosis. In this context, zinc supplementation has been shown to suppress EMT in peritoneal mesothelial cells, at least in part through activation of NRF2 signaling and attenuation of oxidative stress [17,18,22]. Furthermore, salvianolic acid A (Sal A), the major active component of *Salvia miltiorrhiza*, has been shown to enhance NRF2 nuclear translocation and suppress NFκB activation, thereby attenuating peritoneal fibrosis in murine models [19]. Similar results have been observed following treatment with the SGLT-2 inhibitor empagliflozin in HPMCs and rats [20]. A relatively recent murine study by Zhao and colleagues demonstrated that asiaticoside (AC) protects against peritoneal-dialysis-fluid–induced fibrosis by promoting NRF2 nuclear translocation [22]. In addition, a controlled clinical study from Brazil (2023) reported an increase in NRF2 expression in patients with CKD undergoing peritoneal dialysis after two months of treatment with propolis, a resin produced by *Apis mellifera* bees [37].

Clinical evidence for pharmacological NRF2 activation in CKD, however, remains mixed. Treatment with the NRF2 activator bardoxolone methyl was associated with significant increases in eGFR in patients with diabetic CKD in a phase II trial, suggesting potential renoprotective effects [38]. However, the subsequent BEACON phase III trial in patients with type 2 diabetes and stage 4 CKD was terminated early because of an increased risk of heart failure and cardiovascular events. Bardoxolone methyl did not reduce the risk of end-stage renal disease or cardiovascular death [39]. These findings highlight that systemic NRF2 activation may have beneficial effects but may also be associated with clinically relevant adverse effects. Thus, the therapeutic relevance and safety of pharmacological NRF2 activation in CKD remain uncertain.

Taken together, our experimental findings and previous clinical findings support the potential relevance of NRF2-related pathways in the regulation of oxidative stress, inflammation, and fibrosis in the peritoneum. However, the available evidence is largely derived from experimental models or assessments of NRF2 expression rather than direct evaluation of NRF2 transcriptional activity in human peritoneal tissue. Therefore, the reduced NRF2 immunoreactivity observed in our EPS cohort should be interpreted as an association with advanced peritoneal injury rather than as evidence of a causal loss of NRF2 activity. Moreover, because our immunohistochemical assessment did not quantitatively distinguish between nuclear and cytoplasmic NRF2 staining, the present findings cannot determine the extent of NRF2 nuclear translocation or transcriptional activation. Further studies addressing NRF2 subcellular localization and downstream target activation will be necessary to clarify the functional significance of these observations.

### Limitations

The findings of this study should be interpreted considering several limitations. Most importantly, the cross-sectional design restricts the analysis to descriptive observations and precludes conclusions about causal relationships or the molecular mechanisms underlying the observed alterations in NRF2 and NFκB immunoreactivity. However, the present results provide a framework for future research.

In particular, the observed reduction in NRF2 immunoreactivity in EPS patients cannot be interpreted as evidence of a causal loss of NRF2 activity or as a driver of peritoneal membrane injury. Rather, these findings indicate an association between altered NRF2 immunoreactivity and advanced peritoneal injury. Nevertheless, the present results provide a basis for future experimental and prospective studies investigating the functional role of the NRF2–NFκB axis in peritoneal membrane injury.

A further limitation is that NRF2 immunoreactivity was not separately assessed according to its nuclear or cytoplasmic localization. Since nuclear translocation is required for NRF2-mediated transcriptional activity, the present analysis does not allow for conclusions regarding NRF2 nuclear translocation or transcriptional activation. Thus, differences in overall NRF2 immunoreactivity should not be equated with changes in functional NRF2 activity. Future studies incorporating quantitative assessment of NRF2 subcellular localization and downstream target activation will be required to further clarify the functional significance of the observed alterations.

In addition, NRF2 and NFκB immunoreactivity was assessed using a standardized semi-quantitative Histo-Score approach, which may be less sensitive to subtle differences in staining intensity than fully quantitative methods. Although patients with diabetes mellitus and acute peritonitis were excluded to minimize major confounding factors, the potential influence of other clinical and demographic variables cannot be completely excluded.

Despite these limitations, this study constitutes, to our knowledge, one of the largest immunohistochemical investigations of NRF2 and NFκB immunoreactivity in human peritoneal tissue from patients with varying degrees of peritoneal membrane injury. The findings provide a basis for future mechanistic studies exploring the NRF2–NFκB axis as a potential target for preserving peritoneal membrane function during long-term peritoneal dialysis.

## 5. Conclusions

Reduced NRF2 immunoreactivity in EPS is associated with persistent inflammatory alterations and advanced fibrotic remodeling of the peritoneum. From a translational perspective, the findings suggest that alterations in the NRF2–NFκB axis may be involved in the loss of cytoprotective capacity during long-term peritoneal dialysis. However, given the cross-sectional nature of the study and the lack of assessment of NRF2 subcellular localization and transcriptional activity, these findings should be interpreted as associative rather than causal. Further experimental and prospective studies are needed to determine the functional role of the NRF2–NFκB axis in peritoneal membrane injury and EPS.

## Figures and Tables

**Figure 1:**
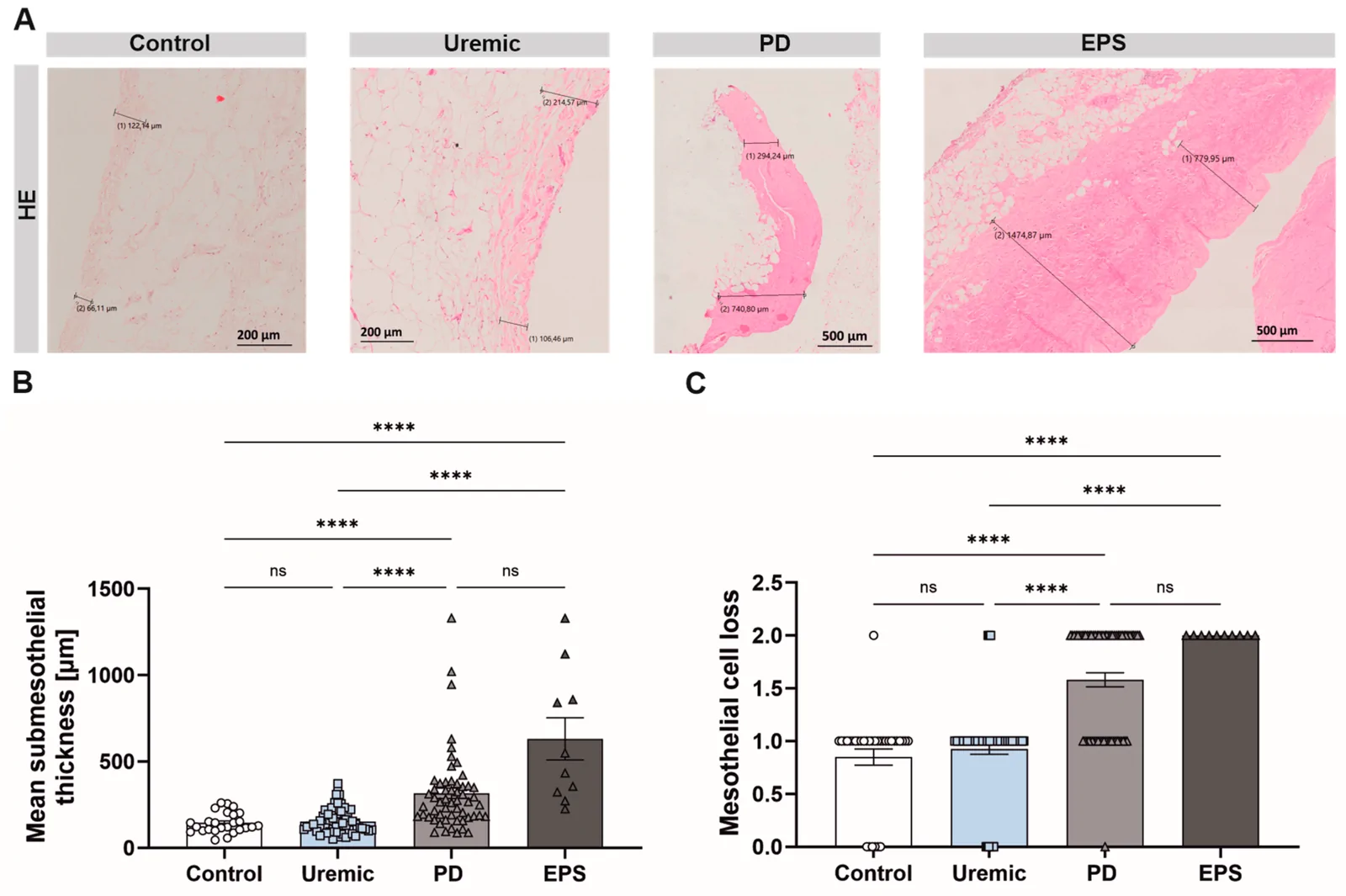
Increases in submesothelial thickness and mesothelial cell loss in patient undergoing PD. (**A**) Representative human peritoneal biopsy sections stained with HE. One representative biopsy from each group is shown (control: n = 34; uremic: n = 69; PD: n = 74 and EPS: n = 14). (**B**) The diagram shows the mean submesothelial thickness across all biopsies for which the measurement could be performed biopsies (mean ± SEM; ****: *p* < 0.0001, and ns: not significant; control: n = 26, uremic: n = 63, PD: n = 56 and EPS: n = 10). (**C**) The diagram shows mesothelial cell loss in peritoneal specimens for which the assessment could be performed (mean ± SEM; ****: *p* < 0.0001, and ns: not significant; control: n = 33, uremic: n = 66, PD: n = 62 and EPS: n = 10). Mesothelial cell loss was scored semi-quantitatively as intact (0), partially absent (1), or completely absent (2). The figure was generated by the authors using published data from Oberacker et al. [25] together with newly collected data from the present study. PD, peritoneal dialysis; EPS, encapsulating peritoneal sclerosis.

**Figure 2:**
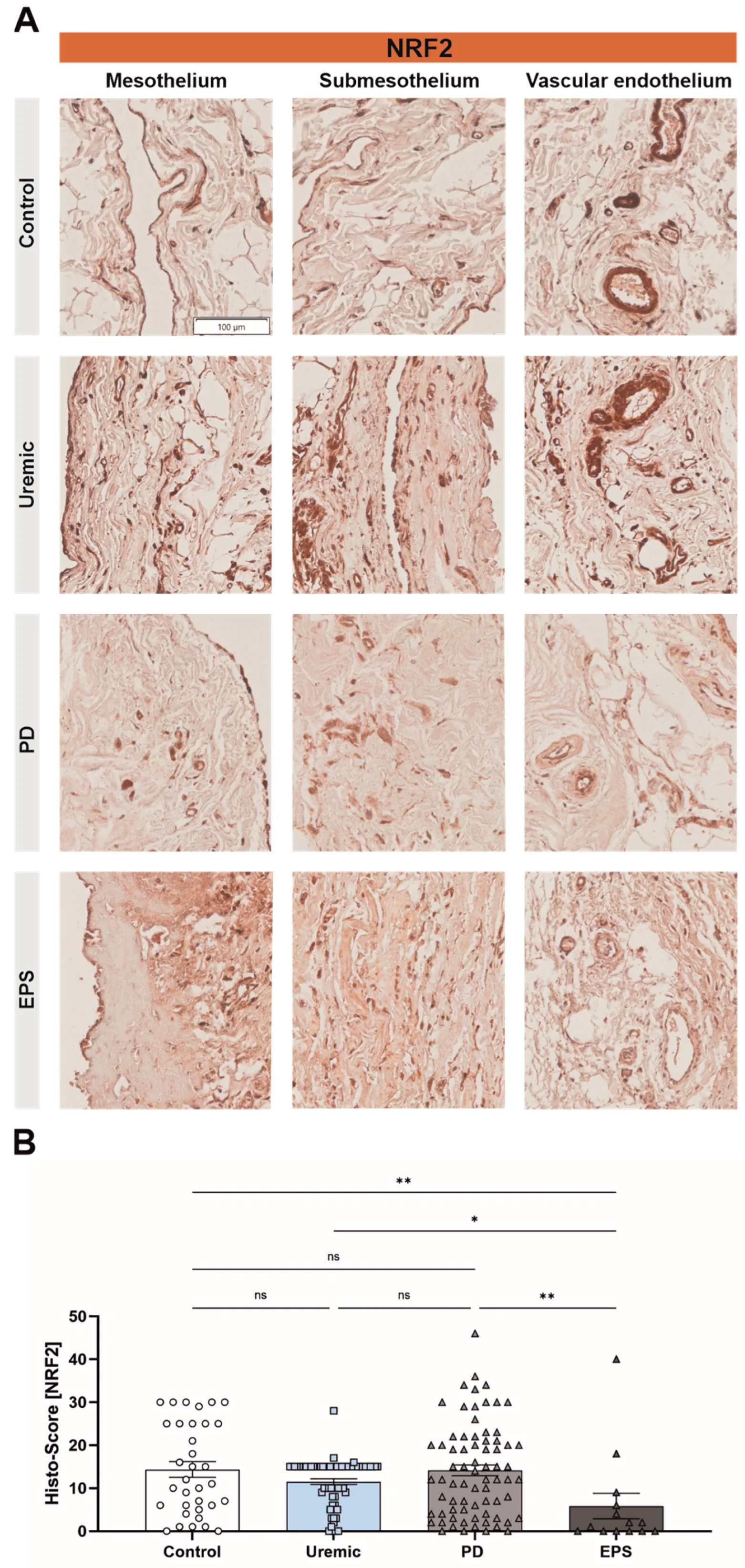
Decreased immunoreactivity of NRF2 in peritoneal biopsies of EPS patients. (**A**) Representative biopsies stained for NRF2. One representative biopsy from each group is shown (control: n = 34, uremic: n = 69, PD: n = 74, EPS: n = 14). Scale bar: 100 µm. (**B**) Quantitative analysis of NRF2 immunoreactivity based on the Histo-Score across all biopsies for which the assessment could be performed (mean ± SEM; **: *p* < 0.01, *: *p* < 0.05, and ns: not significant; control: n = 34, uremic: n = 68, PD: n = 73, and EPS: n = 14). PD: peritoneal dialysis, EPS: encapsulating peritoneal sclerosis.

**Figure 3:**
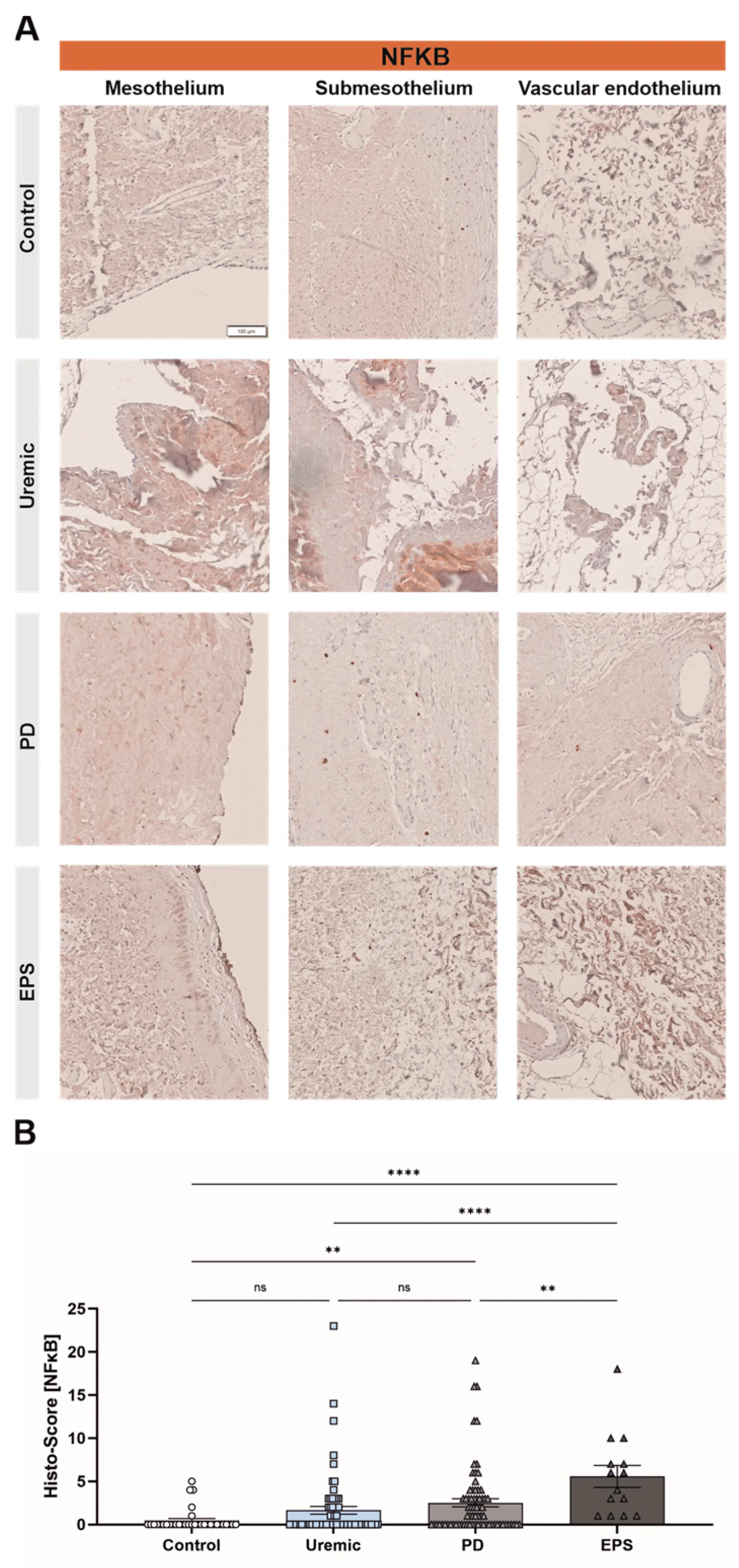
Increased NFκB immunoreactivity in peritoneal biopsies of long-term PD patients. (**A**) Representative biopsies stained for NFκB. One representative biopsy from each group is shown (control: n = 34; uremic: n = 69; PD: n = 74; EPS: n = 14). Scale bar: 100 µm. (**B**) Quantitative analysis of NFκB immunoreactivity based on the Histo-Score across all biopsies for which the assessment could be performed (mean ± SEM; ****: *p* < 0.0001, **: *p* < 0.01, and ns: not significant; control: n = 34, uremic: n = 68, PD: n = 72, and EPS: n = 14). The figure was generated by the authors using published data from Oberacker et al. [25] together with newly collected data from the present study. PD: peritoneal dialysis, EPS: encapsulating peritoneal sclerosis.

**Figure 4:**
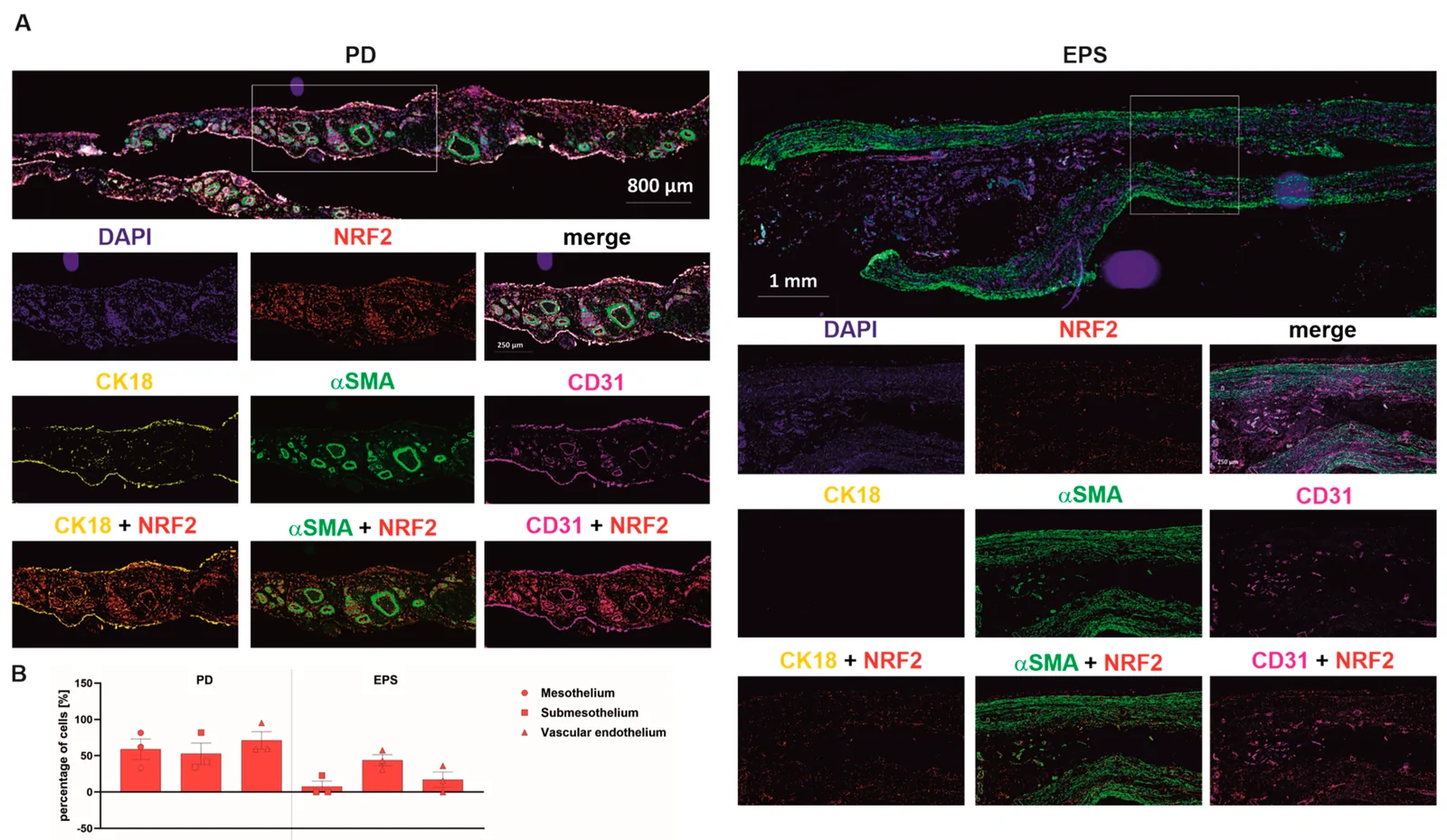
NRF2 staining was observed predominantly within the mesothelial and submesothelial compartments and the vascular endothelium. The extent of mesothelial staining varied between biopsy sections. (**A**) Representative biopsy sections stained for DAPI (blue), NRF2 (red), CK18 (mesothelium, yellow), αSMA (submesothelium, green), and CD31 (vascular endothelium, magenta). (**B**) The bar graphs show the percentage of NRF2-positive cells among all stained cells across the three analyzed biopsy sections (mean ± SEM; *n* = 3).

**Table 1:** Clinical data of study patients. The table was compiled by the authors using published data from Oberacker et al. [15] together with newly collected data from the present study.

	Control	Uremic	PD	EPS
Number (*n*)	34	69	74	14
Age [years], median (IQR)	52.0 (35.80–62.3)	59.0 (46.5–67.0)	57.0 (43.8–65.5)	50.00 (39.8–56.3)
Female/male	27/7	25/44	31/43	4/10
BMI [kg/m^2^], median (IQR)	26.7 (25.6–28.7)	25.6 (21.7–29.4)	25.1 (20.0–29.1)	22.5 (20.4–26.8)
N.D., n	5	2	35	5
Diabetes	Excluded	Excluded	Excluded	Excluded
Hypertension, n (%)	5 (15)	58 (84)	56 (76)	13 (93)
Active smoker, n (%)	5 (15)	5 (7)	9 (12)	2 (14)
Nonsmoker, n (%)	20 (59)	48 (70)	53 (72)	11 (79)
Ex-smoker, n (%)	7 (21)	15 (22)	7 (10)	0 (0)
N.D., n (%)	2 (6)	1 (2)	5 (7)	1 (7)
PD characteristics				
PD duration (months),			32.0 (11.0–53.5)	70.0 (52.8–108.0)
median (IQR)				
PD solutions *				
Glucose, pH-neutral, n (%)			50 (68)	7 (50)
Glucose, pH-acidic, n (%)			18 (24)	3 (21)
Glucose, pH-neutral/acidic, n (%)			3 (4)	1 (7)
Nutrineal, pH-acidic, n (%)			6 (8)	0 (0)
Icodextrin, pH-acidic, n (%)			36 (49)	10 (71)
N.D., n (%)			3 (4)	3 (21)
Transporter status				
High, n (%)			11 (15)	3 (21)
Average, n (%)			27 (37)	7 (50)
Low, n (%)			7 (10)	3 (21)
N.D., n (%)			29 (39)	1 (7)
Laboratory values				
Leucocytes [GIGA/L],				
median (IQR)	6.0 (5.2–7.1)	6.7 (5.7–8.5)	7.2 (5.4–9.4)	6.1 (5.0–8.6)
N.D., n			2	
Hemoglobin [g/L],				
median (IQR)	138.0 (129.8–147.5)	107.0 (95.0–117.0)	110.0 (99.0–125.0)	109.5 (85.8–118.5)
N.D., n			3	
Creatinine [mg/dL],				
median (IQR)	0.76 (0.66–0.87)	6.43 (5.25–8.30)	6.3 (4.0–8.9)	6.2 (4.4–10.4)
N.D., n	1		3	
Glomerular filtration rate (GFR),				
CKD-EPI [mL/min/1.73 m^2^],				
median (IQR)	95.1 (81.3–111.1)	8.0 (6.0–11.0)		
N.D., n	1			
Urea [mg/dL],				
median (IQR)		158.0 (125.5–190.5)	91.9 (53.0–131.5)	71.0 (54.1–112.0)
N.D., n	34		9	1

n: number of values, PD: peritoneal dialysis, IQR: interquartile range, BMI: body mass index, CrP: C-reactive protein, N.D.: not determined, GFR: glomerular filtration rate. * Since one patient receives multiple solutions per day, the sum does not add up to 100.

## Data Availability

The datasets used and/or analyzed during the current study are available from the corresponding author upon reasonable request.

## Supplementary Materials

The following supporting information can be downloaded at https://www.mdpi.com/article/10.3390/antiox15101234/s1: Supplementary Figure S1. Positive and negative controls for immunohistochemical staining of NRF2 and NFκB. Representative images of kidney tissue used as a positive control for NRF2 immunohistochemistry and lymph node tissue used as a positive control for NFκB immunohistochemistry. Corresponding negative controls showed no specific immunoreactivity. Nuclei were counterstained with hematoxylin. Scale bars: 100 µm. Supplementary Figure S2. Rare NRF2 and NFκB immunoreactivity in adipose tissue. Representative adipose tissue sections stained for NRF2 and NFκB. One representative biopsy from each group is shown. For NRF2, the analyzed specimens comprised control (n = 34), uremic (n = 68), PD (n = 73), and EPS (n = 14). For NFκB, the analyzed specimens comprised control (n = 34), uremic (n = 68), PD (n = 72), and EPS (n = 14). Scale bar: 100 µm. PD: peritoneal dialysis; EPS: encapsulating peritoneal sclerosis. Supplementary Figure S3. NRF2 is predominantly localized in the mesothelium, submesothelium, and vascular endothelium. Representative biopsy sections stained for DAPI (blue), NRF2 (red), CK18 (mesothelium, yellow), αSMA (submesothelium, green), and CD31 (vascular endothelium, magenta).

## Abbreviations

The following abbreviations are used in this manuscript:

PD	Peritoneal dialysis
NRF2	Nuclear factor erythroid 2-related factor 2
EPS	Encapsulating peritoneal sclerosis
ELISA	Enzyme-linked immunosorbent assay
NFκB	Nuclear factor kappa-light-chain-enhancer of activated B cells
RRT	Renal replacement therapy
KEAP1	Kelch-like ECH-associated protein 1
HO-1	Heme oxygenase-1
NQO1	NAD(P)H quinone oxidoreductase 1
SOD	Superoxide dismutase
CAPD	Continuous ambulatory peritoneal dialysis
BMI	Body mass index
CrP	C-reactive protein
HE	Hematoxylin and eosin
