# Supplementary material for: Reduced Peritoneal NRF2 Immunoreactivity in Encapsulating Peritoneal Sclerosis and Its Implications for Redox Homeostasis
Source: Antioxidants (Basel). 2026 Sep 24;15(10):1234. doi: 10.3390/antiox15101234 (PMC13700606; doi:10.3390/antiox15101234)
Supplement: Supplementary file 1 [file antioxidants-15-01234-s001.zip › antioxidants-4499889-supplementary.pdf]

Supplementary

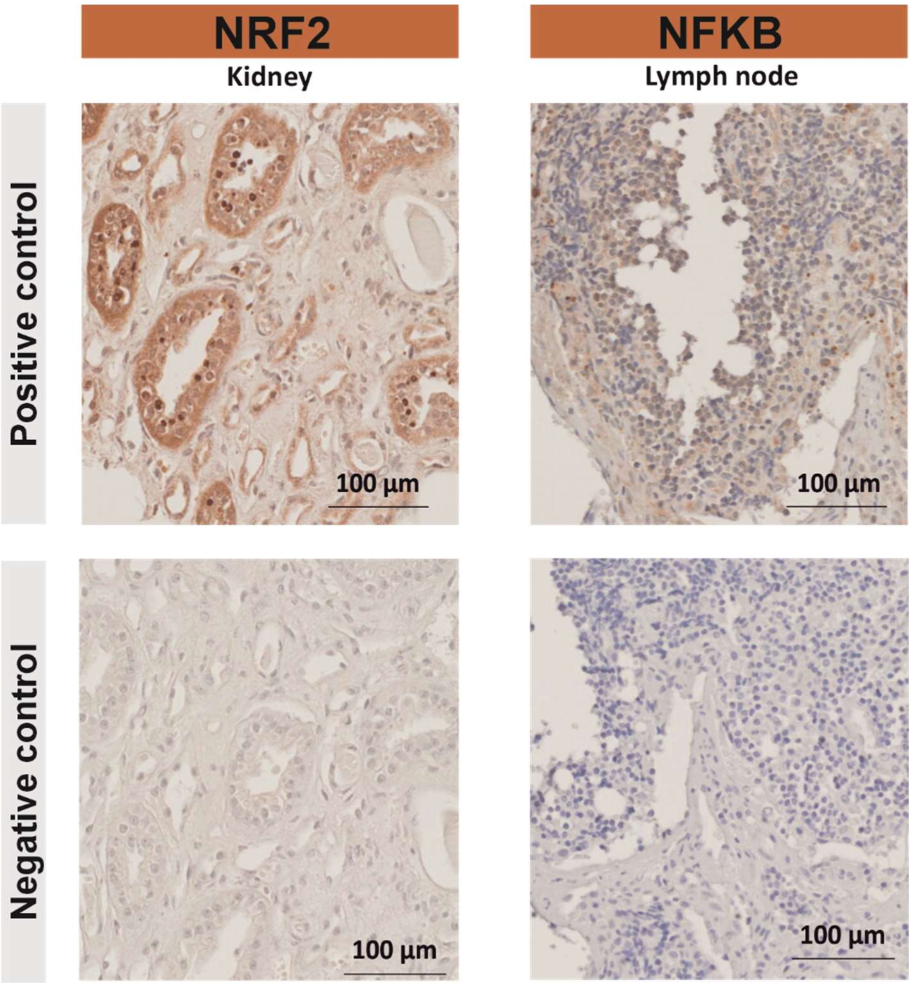

Supplementary Figure S1. Positive and negative controls for immunohistochemical staining of NRF2 and NFκB.

Representative images of kidney tissue used as a positive control for NRF2 immunohistochemistry and lymph node tissue used as a positive control for NFκB immunohistochemistry. Corresponding negative controls showed no specific immunoreactivity. Nuclei were counterstained with hematoxylin. Scale bars: 100 μm.

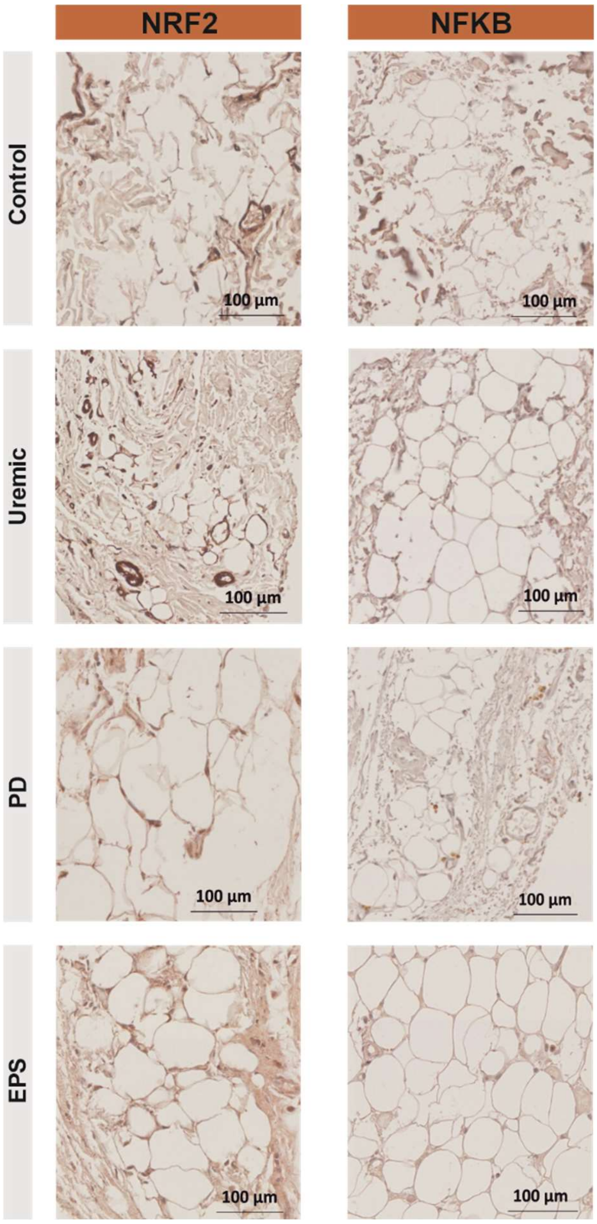

Supplementary Figure S2. **Rare NRF2 and NFκB immunoreactivity in adipose tissue.** Representative adipose tissue sections stained for NRF2 and NFκB. One representative biopsy from each group is shown. For NRF2, the analyzed specimens comprised control (n = 34), uremic (n = 68), PD (n = 73), and EPS (n = 14). For NFκB, the analyzed specimens comprised control (n = 34), uremic (n = 68), PD (n = 72), and EPS (n = 14). Scale bar: 100 µm. PD: peritoneal dialysis; EPS: encapsulating peritoneal sclerosis.

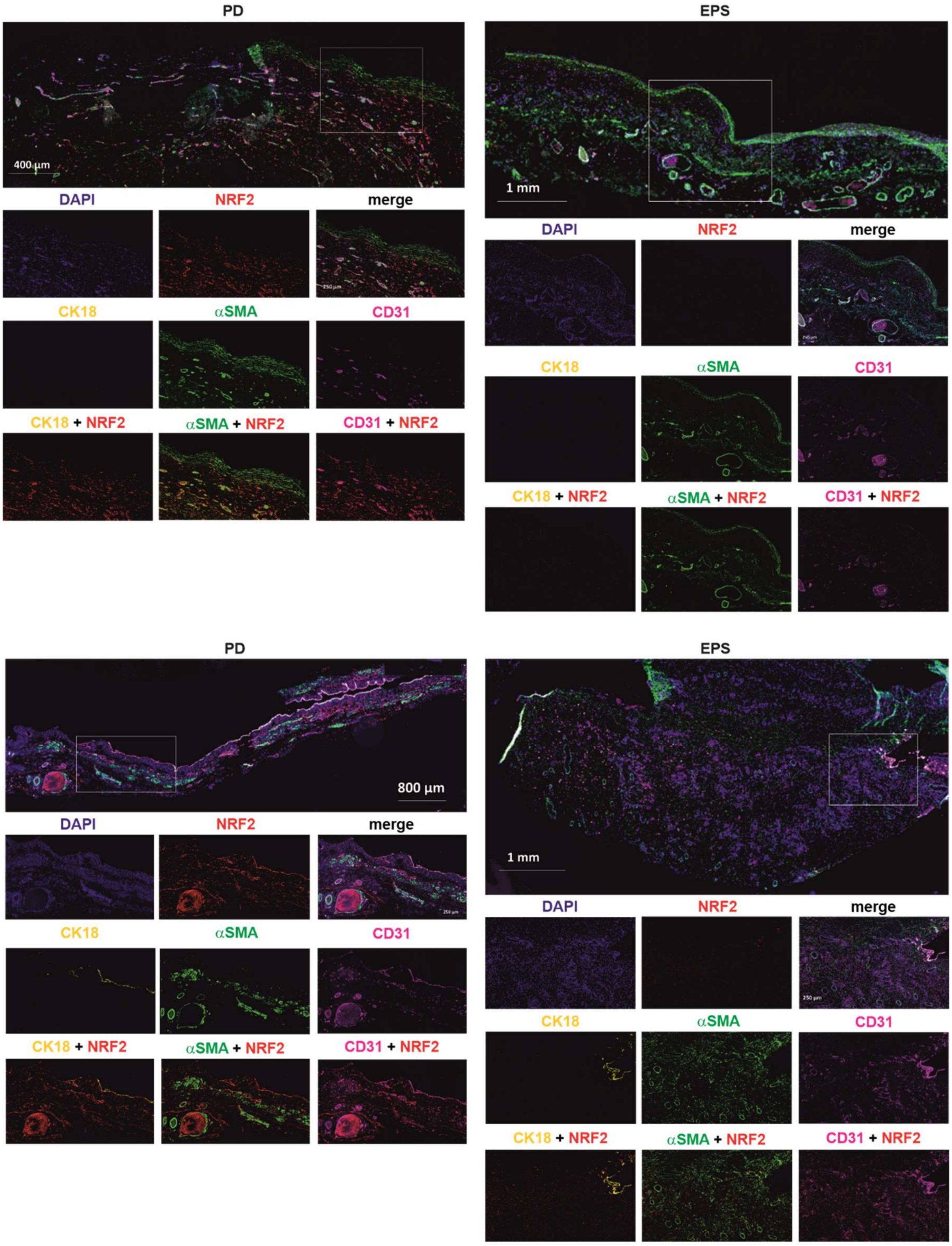

Supplementary Figure S3. NRF2 is predominantly localized in the mesothelium, submesothelium, and vascular endothelium. Representative biopsy sections stained for DAPI (blue), NRF2 (red), CK18 (mesothelium, yellow), αSMA (submesothelium, green), and CD31 (vascular endothelium, magenta).
